# Prevalence, persistence, and severity of 12-month and 30-day DSM-5 disorders in the World Mental Health Hong Kong Study

**DOI:** 10.1016/j.lanwpc.2025.101757

**Published:** 2025-11-26

**Authors:** Corine S.M. Wong, Candi M.C. Leung, Shiyi Wu, Francis P. Flores, Yoona Kim, Xiao Xiao, Solomon B.K. Wong, Wing Chung Chang, Wai Chi Chan, Nancy Xiaonan Yu, Calvin P.W. Cheng, Albert K.K. Chung, Edwin H.M. Lee, Wai Tat Chiu, William G. Axinn, Ronald C. Kessler, Michael Y. Ni

**Affiliations:** a School of Public Health, LKS Faculty of Medicine, The University of Hong Kong, Hong Kong Special Administrative Region, China; b Department of Psychiatry, LKS Faculty of Medicine, The University of Hong Kong, Hong Kong Special Administrative Region, China; c Department of Social and Behavioural Sciences, City University of Hong Kong, Hong Kong Special Administrative Region, China; d Institute for Social Research, University of Michigan, Ann Arbor, MI, United States; e Department of Health Care Policy, Harvard Medical School, Boston, MA, United States; f Urban Systems Institute, The University of Hong Kong, Hong Kong Special Administrative Region, China

**Keywords:** Mental disorder, Prevalence, Epidemiology, Population-based study

## Abstract

**Background:**

The World Mental Health Hong Kong (WMHHK) Study aims to estimate 12-month and 30-day prevalence, persistence, severity, and correlates of DSM-5 anxiety, mood, and externalising disorders in Hong Kong, a densely populated city impacted by consecutive population-level stressors, including social unrest and the COVID-19 pandemic.

**Methods:**

Face-to-face interviews, either in-person or video-based online, were conducted from November 2022 to March 2024 with a population-representative sample of 3053 adults aged 18 years and above. Diagnostic assessment utilised the World Mental Health Composite International Diagnostic Interview for DSM-5 (CIDI-5), evaluating ten mental disorders: anxiety (panic disorder, generalised anxiety disorder, post-traumatic stress disorder, obsessive-compulsive and related disorders), mood (major depressive disorder, persistent depressive disorder, bipolar spectrum disorders), and externalising (intermittent explosive disorder, alcohol use disorder, substance use disorder) disorders. Persistence was defined as 12-month prevalence among lifetime cases and 30-day prevalence among 12-month cases. Sociodemographic correlates were analysed using multivariable logistic regression.

**Findings:**

Twelve-month and 30-day prevalence of any DSM-5 mental disorder were 10.6% (95% CI: 9.5–11.8) and 7.8% (95% CI: 6.7–8.9), respectively. Twelve-month prevalence was highest for anxiety disorders (8.0%, 95% CI: 7.1–8.9), followed by mood (4.3%, 95% CI: 3.4–5.2) and externalising (1.7%, 95% CI: 0.9–2.4) disorders. Twelve-month persistence among lifetime cases was 49.0%, overall and higher for anxiety (55.6%) than mood (39.0%) or externalising (35.3%) disorders. Younger and middle-aged adults, and who were not currently married, had elevated risks, while lower education was associated with greater disorder severity. Comorbidity was associated with increased persistence and severity across disorders.

**Interpretation:**

This study shows a substantial mental health burden in Hong Kong during the post-pandemic period, highlighting the need for tailored public mental health programmes to address urban stressors in this unique context.

**Funding:**

10.13039/501100013947WYNG Foundation, Hong Kong; 10.13039/501100024334State Key Laboratory of Brain and Cognitive Sciences, The 10.13039/501100003803University of Hong Kong, Hong Kong Special Administrative Region, China; Hong Kong Jockey Club Charities Trust.

**Research in context**

**Evidence before this study**

We conducted a comprehensive review of population-based studies examining mental disorders during the endemic and post-COVID-19 period through a search of PubMed, Web of Science, and PsycINFO from database inception to July 17, 2025. Our search strategy used the following terms with no language restrictions in PubMed: ((“population-based” [MeSH] OR “epidemiologic studies” [MeSH] OR “epidemiologic” [All Fields] OR “epidemiological” [All Fields])) AND (“prevalence” [MeSH]) AND ((“mental disorders” [MeSH] OR “mood disorders” [MeSH] OR “depressive disorder” [MeSH] OR “anxiety disorders” [MeSH] OR “substance use disorders” [All Fields] OR “alcohol use disorders” [All Fields] OR “externalising disorder” [All Fields])) AND ((“endemic” [All Fields] OR “post-COVID” [All Fields] OR “post-pandemic” [All Fields])). The search string was adapted for other databases. To our knowledge, existing population-based studies reporting prevalence estimates of mental disorders during and after COVID period have relied on screening instruments. No studies to date have employed structured diagnostic interviews in representative population samples to establish clinically validated prevalence rates in the post-pandemic era.

**Added value of this study**

The World Mental Health Hong Kong (WMHHK) Study provides the first population-representative DSM-5 diagnostic estimates of mental disorders in Hong Kong, using the gold-standard Composite International Diagnostic Interview (CIDI-5) with a stratified random sample of 3053 adults. We assessed the prevalence, persistence, severity, and correlates of a wide range of mental disorders during the endemic and post-pandemic period (2022–2024). This investigation reveals a significant mental health burden in Hong Kong. One in ten adults reported having at least one mental disorder in the past 12 months, with 28.5% classified as severe cases and 49.0% showing symptom persistence among lifetime cases. Anxiety disorders were the most prevalent (8.0%) and persistent (55.6%), while mood disorders and externalising disorders affected 4.3% and 1.7% of respondents, respectively. Younger and middle-aged adults, as well as unmarried individuals, were associated with higher risks of mental disorders, while respondents with lower education showed higher odds of having severe disorders. Comorbidity was linked to both persistence and severity across all disorders.

**Implications of all the available evidence**

Mental disorders represent a critical public health challenge worldwide, significantly contributing to the global burden of disease and disability. Despite their high prevalence and impact, they often remain underdiagnosed and undertreated, necessitating timely epidemiologic surveys to understand mental health burdens, particularly following major population events. The WMHHK is the most up-to-date territory-wide cohort study in Hong Kong. It is also one of the few population-based studies conducted in the post-pandemic period. We identified a major mental health burden, marked by high persistence and severity, in this densely populated urban metropolis that has endured a series of impactful events, including social unrest and the COVID-19 pandemic. Our findings offer critical insights for developing targeted interventions for populations affected by these major events, providing an empirical framework for future research on the long-term effects on vulnerable groups. In particular, younger and middle-aged adults, as well as unmarried or less-educated individuals, were more likely to be affected, highlighting specific risk profiles. These insights will facilitate evidence-based policymaking to enhance population mental health, emphasising the need for tailored strategies that address both immediate and sustained mental health challenges in high-stress urban contexts like Hong Kong, while informing global approaches to post-crisis population mental health.

## Introduction

Over the past few years, many countries have been impacted by consecutive major population-level stressors, including the COVID-19 pandemic, conflicts, major protests, and natural disasters. These events have likely exacerbated psychological distress in the population, which could be examined by focusing on a series of mental disorders found to be commonly occurring and seriously impairing.^1^^,^^2^ Mental disorders account for approximately 13% of the global burden of disease, with mood and anxiety disorders being particularly prevalent and among the leading causes of years lived with disability.^3^^,^^4^ The impact extends beyond individuals, affecting relationships, productivity, and imposing substantial societal burdens on healthcare systems, economies, and communities.^5^ However, data on population mental health are lacking in the post-pandemic period, leaving the magnitude of health needs and vulnerable groups unclear.

Epidemiologic surveys have been instrumental in advancing our understanding of mental disorders across diverse populations over recent decades. Despite their high prevalence and associated burden, mental disorders often remain underdiagnosed and undertreated, especially in regions where stigma, limited resources, and lack of awareness create significant barriers to care.^6^ Epidemiologic surveys provide crucial data in this context on prevalence, persistence, severity, and treatment patterns of mental disorders. Such data are essential for shaping public health policies, resource allocation, and targeted interventions for prevention and treatment.^7^ The World Mental Health (WMH) Surveys, conducted in over 30 countries, enable cross-national comparisons of mental disorders through standardised diagnostic tools, while also identifying local challenges and informing the design of culturally relevant strategies.^8^, ^9^, ^10^

Hong Kong, having experienced both the 2019 social unrest and the COVID-19 pandemic consecutively, serves as an epidemiologic sentinel for examining the combined effects of major population events on mental health in the post-pandemic period.^11^ As a densely populated, urbanised, and fast-paced metropolis with over 7.5 million residents, Hong Kong shares characteristics with many other highly developed Asian economies (e.g., Singapore, South Korea, Japan), including urban density, socioeconomic pressures, and long working hours, alongside social isolation and loneliness intensified by the public health and social measures implemented during the pandemic.^12^^,^^13^ All these factors potentially contribute to mental disorders.^14^, ^15^, ^16^ Understanding the mental health landscape in Hong Kong can therefore offer valuable insights for other highly developed economies facing comparable challenges. Previous epidemiologic studies of mental disorders in Hong Kong have often focused on specific disorders, subpopulations, or clinical settings, or relied on non-probability sampling, limiting a holistic view of the mental health landscape. The most recent territory-wide mental health survey (Hong Kong Mental Morbidity Survey, HKMMS) was conducted over a decade ago,^17^^,^^18^ leaving a significant gap in comparable data on the current state of mental health across the general population. To guide policy-informed interventions, priority setting, and service planning, there is a pressing need for comprehensive, standardised, and up-to-date data from a population-representative survey.

Accordingly, the World Mental Health Hong Kong (WMHHK) Study was conducted from 2022 to 2024 as part of the WMH Surveys to provide population-based estimates of mental disorders in Hong Kong. In this study, mental disorders were assessed with the Composite International Diagnostic Interview (CIDI-5)^7^ to operationalise diagnostic criteria according to the Diagnostic and Statistical Manual of Mental Disorders, 5th Edition (DSM-5).^19^ We present data on 12-month and 30-day prevalence (cases meeting diagnostic criteria in the past 12 months and past 30 days), 12-month persistence (12-month prevalence among lifetime cases) and 30-day persistence (30-day prevalence among 12-month cases), severity (mild, moderate, or severe, according to the WMH three-tier classification scheme),^10^ and comorbidity (number of concurrent 12-month or 30-day disorders). We also examined sociodemographic correlates of prevalence and persistence. This marks the first report from WMHHK. Subsequent reports will focus on lifetime prevalence, age of onset, treatment, and other topics.

## Methods

### Study design and participants

Our sample was randomly drawn from the FAMILY Cohort, a prospective population-representative cohort study in Hong Kong.^11^^,^^20^ The baseline Cohort sample, recruited in 2009–2011, was obtained through stratified random sampling of households across all 18 districts, with additional purposive oversampling in three new towns.^20^ These towns were selected for their relative remoteness and higher concentration of newly arrived mainland Chinese migrants, and were oversampled to enhance the population representativeness of the cohort.^20^ Sampling frames for each district were constructed using complete listings of living quarters in Hong Kong. The data collected include socio-demographic characteristics, lifestyle and behavioural factors, physical and mental health indicators, as well as household- and neighbourhood-level characteristics. The WMHHK was conducted between November 2022 and March 2024, during which the CIDI-5 was administered to 3053 eligible adult participants (aged ≥18 years) within the FAMILY Cohort. Computer-assisted, face-to-face interviews were conducted in real time by trained and certified interviewers using two modes: (1) in-person or (2) video-based online. Interview content and instruments were consistent across both. Due to COVID-19 restrictions and to maximise participation, most interviews were conducted via online video (n = 2927; 95.9%), with the others conducted in person (n = 126; 4.1%). Regular quality control was performed in accordance with the WMH Quality Assurance Guidelines to ensure data reliability (Appendix p 2–4). Written informed consent was obtained from all participants. The study was approved by the Institutional Review Board of the University of Hong Kong/Hospital Authority Hong Kong West Cluster (Reference no.: UW 20–727).

### Survey instrument

Diagnostic assessments were conducted using the fully structured, lay-administered CIDI-5, an updated version of the CIDI 3.0 revised to meet the DSM-5 criteria.^7^^,^^19^ We developed the Traditional Chinese adaptation of CIDI-5 through a multi-phase process, including expert panel review by psychiatrists and psychologists, cultural adaptation, pre-testing, and cognitive interviewing (Appendix p 2). Diagnostic validity was established through concordance testing against the gold-standard Structured Clinical Interview for DSM-5 (SCID-5).^21^ All assessments were administered by staff with mental health expertise who were trained and closely supervised by psychiatrists. We validated major depressive disorder (MDD), generalised anxiety disorder (GAD), and post-traumatic stress disorder (PTSD), observing excellent psychometric properties, including excellent test-retest reliability (α = 0.89–0.93), validity (κ = 0.82–0.91), and good concordance with independent clinical diagnoses based on the SCID-5 (AUC-ROC = 0.881, 0.861, and 0.820 for MDD, GAD, and PTSD, respectively; sTable 1).

We assessed 10 mental disorders, including (1) four anxiety disorders (panic disorder, GAD, PTSD, and obsessive-compulsive and related disorders [OCRD]); (2) three mood disorders (MDD, persistent depressive disorder [PDD], and bipolar spectrum disorders [BPS]); and (3) three externalising disorders (intermittent explosive disorder [IED], alcohol use disorder [AUD], and substance use disorder [SUD]) (sTable 2). These disorders are WMH CIDI-5 core disorders and were selected based on previous evidence of their joint frequency and severity in the general population.^7^ Disorders that typically require specialised clinical assessment (e.g., schizophrenia spectrum and other psychotic disorders, personality disorders) were excluded due to time constraints and limited diagnostic accuracy in population-based surveys.^22^, ^23^, ^24^, ^25^ While DSM-5 reclassified PTSD and OCRD into separate diagnostic categories, we maintained their grouping under anxiety disorders to ensure consistency for comparisons with other countries with prior DSM-IV classifications. We also grouped IED, AUD, and SUD to represent the spectrum of externalising behaviours. We report 12-month and 30-day prevalence following WMH conventions to maximise cross-national comparability. Twelve-month estimates inform annual service planning and burden estimation while maintaining a feasible recall period,^10^ while 30-day estimates provide timely indicators of current impairment and service needs with less recall bias.^7^ Comorbidity was defined as the number of concurrent disorders (1, 2, or ≥3). We defined 12-month persistence as the ratio of 12-month prevalence to lifetime prevalence, and 30-day persistence as the ratio of 30-day prevalence to 12-month prevalence.^26^^,^^27^ Severity was categorised as mild, moderate, or severe using the standard three-tier system in the WMH Survey Initiative^10^ (Appendix p 4).

Sociodemographic characteristics included sex, age, marital status, and employment status. Educational attainment was classified into four levels: low (primary education or below), low-average (secondary education or post-secondary diploma), high-average (associate degree or bachelor's degree), and high (master's degree or above). Income was categorised into low, low-average, high-average, and high, based on the ratio of income per capita to the median income (low defined as less than half the median, low-average as up to the median, high-average as up to two times the median, and high as more than two times the median).

### Statistical analysis

A weight based on inverse probability of participation was used to adjust for attrition bias.^28^ This weight was derived using logistic regression estimates of the likelihood of participating in the study, based on sociodemographic characteristics assessed in the baseline waves of the FAMILY Cohort.^29^ Raking was then applied to ensure that the sample would be representative of the general population in Hong Kong^30^ (Appendix p 4).

We estimated 12-month and 30-day prevalence with these weighted data, generating separate estimates for any disorder, three disorder categories (anxiety, mood, and externalising disorders), and each individual disorder. The models for persistence were estimated by assessing 12-month prevalence among lifetime cases and 30-day prevalence among 12-month cases. Severity was assessed for both 12-month and 30-day disorders. Prevalence estimates were reported with 95% confidence intervals (95% CIs). To evaluate the potential effects of interview mode, we conducted sensitivity analyses using machine-learning models for robust confounding adjustment. For each 12-month outcome, we trained a random forest on all sociodemographic covariates to generate respondent-level risk scores, then fitted logistic regressions that included the risk score and interview mode (in-person vs. video-based online). Interview mode was not significantly associated with any outcome, indicating no differences in prevalence estimates by interview mode (Appendix p 4). Sociodemographic correlates of 12-month and 30-day prevalence, persistence, and severity were examined using logistic regression. In addition to grouping PTSD and OCRD with anxiety disorders for overall prevalence comparisons, analyses were also performed treating PTSD and OCRD as distinct categories for sociodemographic correlates. Standard errors of estimates were obtained using the Taylor series linearisation method implemented in SAS version 9.4 (SAS Institute, Cary, NC). Logit estimates and their 95% CIs, calculated as the estimate ± t (0.975, df) × standard error, were exponentiated to generate odds ratios (ORs) and corresponding design-based 95% CIs. Multivariable significance tests of predictor sets were conducted using Wald F-tests with Taylor series design-based coefficient variance-covariance matrices. All significance tests were evaluated at α = 0.05 with two-sided tests.

### Role of funding sources

The funders of the study had no role in the design and conduct of the study; collection, management, analysis, and interpretation of the data; preparation, review, and approval of the manuscript; or the decision to submit the manuscript for publication.

## Results

A total of 3053 respondents completed the study. The response and cooperation rate were 25.5% and 48.4%, respectively. The weighted sample showed small to medium sociodemographic differences compared to Hong Kong's 2021 census population in sex, age, education, household income, employment, and marital status (Cohen's *w* effect size 0.01–0.24; Table 1).

**Table 1:** Sociodemographic characteristics of WMHHK participants compared with the population of Hong Kong.

	WMHHK (unweighted)	WMHHK (weighted)	2021 Hong Kong population census	WMHHK (weighted) vs. census
	n (%)	n (%)	n (%)	Effect size
**Sex**				
Female	1690 (55.4)	1619 (53.0)	3,242,961 (52.9)	0.002
Male	1363 (44.6)	1434 (47.0)	2,882,441 (47.1)	
**Age (years)**				
18–34	648 (21.2)	732 (24.0)	1,322,163 (21.6)	0.103
35–49	633 (20.7)	673 (22.0)	1,564,958 (25.5)	
50–64	1094 (35.8)	965 (31.6)	1,787,423 (29.2)	
65 and above	678 (22.2)	683 (22.4)	1,450,858 (23.7)	
**Education**^a^				
Low	421 (13.8)	596 (19.5)	1,499,633 (24.9)	0.237
Low average	1709 (56.0)	1599 (52.4)	2,443,897 (40.6)	
High average or high^b^	923 (30.2)	858 (28.1)	2,076,817 (34.5)	
**Household income (HKD)**				
<10,000	657 (21.5)	767 (25.1)	534,147 (20.0)	0.143
10,000–19,999	389 (12.7)	529 (17.3)	485,017 (18.2)	
20,000–39,999	843 (27.6)	832 (27.2)	702,410 (26.3)	
≥40,000	1164 (38.1)	925 (30.3)	950,110 (35.6)	
**Employment status**				
Others^c^	1021 (33.4)	1074 (35.2)	2,685,376 (44.6)	0.197
Working	2032 (66.6)	1979 (64.8)	3,334,971 (55.4)	
**Marital status**				
Never married	730 (23.9)	783 (25.7)	1,651,322 (27.0)	0.048
Previously married^d^	356 (11.7)	367 (12.0)	796,694 (13.0)	
Married	1967 (64.4)	1903 (62.3)	3,677,386 (60.0)	

Cohen's w effect size, small 0.1; medium 0.3; large 0.5. Inverse probability of censoring weighting and raking are used. HKD, Hong Kong Dollar.

a Educational attainment is defined as low (primary education or below), low-average (secondary education or post-secondary diploma), high-average (associate degree or bachelor's degree), and high (master's degree or above).

b Post-secondary education (including diploma, associate degree, bachelor's degree, and master's degree or above) is grouped into “high average or high” education due to the unavailability of data for separate categories in the 2021 Hong Kong Population Census.

c Others include students, homemakers, retirees and others.

d Previously married includes separated, divorced and widowed individuals.

The 12-month prevalence of any DSM-5 mental disorder was 10.6% (95% CI: 9.5–11.8), with anxiety disorders (8.0%, 95% CI: 7.1–8.9) being the most common, followed by mood disorders (4.3%, 95% CI: 3.4–5.2) and externalising disorders (1.7%, 95% CI: 0.9–2.4). The three individual disorders with the highest prevalence were OCRD (3.7%, 95% CI: 3.1–4.4), PTSD (3.4%, 95% CI: 2.7–4.0), and MDD (2.8%, 95% CI: 2.1–3.5). SUD was reported with zero prevalence (Table 2). Of the total sample, 6.7% experienced one disorder, 2.2% had two, and 1.8% had three or more disorders in the past 12 months. The 12-month cases represent a 49.0% persistence among lifetime cases, with anxiety disorders (55.6%) having higher persistence than mood disorders (39.0%) and externalising disorders (35.3%). Specifically, BPS exhibited the highest persistence (64.2%), whereas AUD had the lowest (31.8%). Twelve-month persistence was highly related to lifetime comorbidity, with 80.6% of respondents who reported three or more lifetime disorders having at least one 12-month disorder (Table 2).

**Table 2:** Twelve-month and 30-day prevalence and persistence of DSM-5 disorders in the WMHHK (N = 3053).

	Prevalence		Persistence	
	12-month % (95% CI)	30-day % (95% CI)	12-month % (95% CI)^a^	30-day % (95% CI)^b^
Anxiety disorders				
Panic disorder	1.0 (0.6–1.5)	0.8 (0.3–1.3)	57.7 (43.4–72.0)	74.4 (55.1–93.8)
Generalised anxiety disorder	2.1 (1.5–2.6)	1.7 (1.2–2.2)	52.3 (42.1–62.6)	81.9 (70.0–93.9)
Post-traumatic stress disorder	3.4 (2.7–4.0)	2.5 (2.0–3.0)	43.9 (37.7–50.2)	73.4 (64.5–82.4)
Obsessive-compulsive and related disorders	3.7 (3.1–4.4)	3.2 (2.6–3.8)	61.2 (53.0–69.3)	84.8 (75.6–94.1)
Any anxiety disorder^c^	8.0 (7.1–8.9)	6.4 (5.6–7.2)	55.6 (50.9–60.3)	79.9 (72.6–87.1)
Mood disorders				
Major depressive disorder	2.8 (2.1–3.5)	1.1 (0.5–1.7)	34.4 (28.5–40.2)	38.4 (19.7–57.2)
Persistent depressive disorder	2.0 (1.3–2.7)	1.1 (0.6–1.5)	49.4 (39.3–59.5)	53.0 (35.2–70.8)
Bipolar spectrum disorders	0.5 (0.1–0.9)	0.3 (0.0–0.6)	64.2 (45.0–83.5)	58.6 (28.0–89.2)
Any mood disorder	4.3 (3.4–5.2)	2.0 (1.4–2.5)	39.0 (34.7–43.2)	45.9 (36.0–55.9)
Externalising disorders^d^				
Intermittent explosive disorder	0.5 (0.2–0.8)	0.3 (0.2–0.5)	57.7 (28.9–86.6)	65.6 (42.5–88.6)
Alcohol use disorder	1.2 (0.5–1.8)	0.9 (0.4–1.4)	31.8 (15.6–48.1)	80.8 (65.0–96.7)
Any externalising disorder	1.7 (0.9–2.4)	1.3 (0.7–1.8)	35.3 (20.3–50.3)	76.2 (62.6–89.8)
Number of disorders				
One disorder	6.7 (5.7–7.7)	5.3 (4.2–6.4)	35.2 (31.2–39.2)^e^	61.2 (52.5–69.9)^f^
Two disorders	2.2 (1.6–2.8)	1.7 (1.3–2.2)	57.7 (46.2–69.3)^e^	92.7 (87.1–98.3)^f^
Three or more disorders	1.8 (1.2–2.3)	0.8 (0.4–1.2)	80.6 (72.1–89.1)^e^	98.5 (96.2–100.0)^f^
Any mental disorder	10.6 (9.5–11.8)	7.8 (6.7–8.9)	49.0 (44.2–53.8)^e^	73.8 (67.1–80.6)^f^

CI, confidence interval.

a The 12-month persistence is calculated as the 12-month prevalence among lifetime cases.

b The 30-day persistence is calculated as the 30-day prevalence among 12-month cases.

c The 12-month and 30-day prevalence of any anxiety disorder (excluding generalised anxiety disorder) is 6.8% and 5.3%, respectively.

d Substance use disorder is reported with zero prevalence.

e Cases with at least one 12-month mental disorder, stratified by number of (1, 2, ≥3, or any) lifetime disorder(s).

f Cases with at least one 30-day mental disorder, stratified by number of (1, 2, ≥3, or any) 12-month disorder(s).

Among respondents with any 12-month disorder, 28.5% were classified as having a severe disorder, 45.9% as moderate, and 25.6% as mild (Table 3). Notably, individuals with three or more 12-month disorders showed the greatest severity, with 50.4% classified as severe and 48.5% as moderate. Compared with those with a single 12-month disorder, respondents with two disorders were twice as likely to be classified as severe (39.3% vs. 19.2%; p = 0.049), and those with three or more disorders had 2.6 times the odds of being classified as severe (50.4% vs. 19.2%; p < 0.001) (Table 3). Mild cases were predominantly single-disorder cases (96.8%), with only 2.5% having two disorders and 0.7% having three or more (sTable 3).

**Table 3:** Severity distribution for 12-month and 30-day DSM-5 disorders in the WMHHK.

	12-month disorder^a^^,^^b^			30-day disorder^a^^,^^c^		
	Severe % (95% CI)	Moderate % (95% CI)	Mild % (95% CI)	Severe % (95% CI)	Moderate % (95% CI)	Mild % (95% CI)
Anxiety disorders						
Panic disorder	37.9 (17.3–58.4)	41.8 (21.7–61.8)	20.4 (3.8–36.9)	31.4 (10.4–52.5)	43.4 (23.6–63.1)	25.2 (2.9–47.5)
Generalised anxiety disorder	54.6 (41.9–67.3)	45.1 (32.9–57.3)	0.3 (0.0–0.9)	64.0 (51.2–76.8)	36.0 (23.2–48.8)	0.0
Post-traumatic stress disorder	29.8 (16.5–43.2)	58.9 (48.8–69.0)	11.3 (1.5–21.1)	33.0 (16.9–49.2)	57.6 (46.4–68.8)	9.4 (0.0–20.5)
Obsessive-compulsive and related disorders	17.2 (6.7–27.7)	34.3 (25.4–43.1)	48.5 (36.9–60.1)	17.6 (8.4–26.8)	36.7 (28.3–45.1)	45.7 (36.7–54.7)
Any anxiety disorder	25.6 (16.3–34.9)	45.6 (39.7–51.5)	28.8 (21.2–36.4)	27.7 (18.4–36.9)	44.2 (37.9–50.6)	28.1 (21.1–35.1)
Mood disorders						
Major depressive disorder	44.3 (30.3–58.2)	52.6 (38.9–66.3)	3.1 (0.0–7.9)	70.2 (56.9–83.6)	29.8 (16.4–43.1)	0.0
Persistent depressive disorder	31.5 (12.7–50.4)	60.3 (42.2–78.4)	8.2 (0.0–18.7)	47.5 (20.0–75.0)	52.5 (25.0–80.0)	0.0
Bipolar spectrum disorders	49.2 (22.0–76.5)	41.6 (15.8–67.3)	9.2 (0.0–27.9)	77.3 (43.8–100.0)	6.9 (0.0–21.9)	15.7 (0.0–46.5)
Any mood disorder	38.6 (29.4–47.8)	54.4 (43.6–65.1)	7.0 (0.7–13.4)	58.5 (41.9–75.2)	39.0 (22.2–55.8)	2.4 (0.0–7.5)
Externalising disorders^d^						
Intermittent explosive disorder	37.4 (19.4–55.4)	62.6 (44.6–80.6)	0.0	51.3 (28.6–74.0)	48.7 (26.0–71.4)	0.0
Alcohol use disorder	73.1 (57.7–88.6)	16.9 (3.6–30.2)	10.0 (0.0–21.8)	71.3 (50.0–92.7)	16.3 (0.0–34.5)	12.3 (0.0–26.3)
Any externalising disorder	62.3 (46.7–77.9)	30.7 (16.9–44.6)	7.0 (0.0–15.4)	66.1 (46.9–85.4)	24.7 (8.1–41.4)	9.1 (0.0–19.9)
Number of disorders						
One disorder	19.2 (12.2–26.2)	41.4 (33.6–49.2)	39.4 (32.2–46.7)	24.4 (15.7–33.2)	40.1 (32.6–47.7)	35.5 (26.8–44.1)
Two disorders	39.3 (20.0–58.5)	57.6 (39.6–75.5)	3.1 (0.0–7.6)	37.2 (21.2–53.1)	58.0 (42.7–73.4)	4.8 (0.0–11.3)
Three or more disorders	50.4 (38.0–62.8)	48.5 (35.6–61.4)	1.1 (0.0–3.5)	76.3 (62.3–90.3)	23.7 (9.7–37.7)	0.0
Any mental disorder	28.5 (21.8–35.2)	45.9 (39.8–52.0)	25.6 (19.7–31.5)	32.8 (24.7–40.8)	42.3 (36.0–48.5)	25.0 (18.7–31.2)

CI, confidence interval.

a Percentages indicate the distribution of each severity category by disorder.

b A significant difference in disorder severity by number of 12-month disorders is observed (F (4,13) = 46.8, p < 0.001). Respondents with two comorbid disorders are significantly more likely to be serious compared to those with only one disorder (F (1,18) = 4.4, p = 0.049). Respondents with three or more disorders are not significantly more likely to be serious compared to those with two disorders (F (1,18) = 1.3, p = 0.27). Respondents with three or more disorders are significantly more likely to be serious compared to those with only one disorder (F (1,18) = 21.6, p < 0.001).

c A significant difference in disorder severity by number of 30-day disorders is observed (F (2,12) = 24.2, p < 0.001). The test with 2 df is used because of the empty cell for mild severity and three or more 30-day disorders. The two disorders and three or more disorders categories are combined to ensure model stability and enable valid statistical testing. Respondents with two comorbid disorders are not significantly more likely to be serious compared to those with only one disorder (F (1,18) = 2.4, p = 0.14). Respondents with three or more disorders are significantly more likely to be serious compared to those with two disorders (F (1,18) = 10.1, p = 0.005). Respondents with three or more disorders are significantly more likely to be serious compared to those with only one disorder (F (1,18) = 29.8, p < 0.001).

d Substance use disorder is reported with zero prevalence.

The 30-day prevalence of any mental disorder, anxiety disorder, mood disorder, and externalising disorder were 7.8% (95% CI: 6.7–8.9), 6.4% (95% CI: 5.6–7.2), 2.0% (95% CI: 1.4–2.5), and 1.3% (95% CI: 0.7–1.8), respectively. Among those with any 12-month disorder, nearly three-quarters (73.8%) experienced symptoms in the past 30 days, with higher 30-day persistence for anxiety (79.9%) than externalising (76.2%) or mood (45.9%) disorders. Notably, OCRD had the highest 30-day persistence (84.8%) and MDD had the lowest (38.4%). Among respondents with three or more 12-month disorders, nearly all (98.5%) had at least one 30-day disorder (Table 2). Severe 30-day cases were found in 77.3% of respondents with BPS, 71.3% with AUD, and 70.2% with MDD (Table 3). Among all severe cases, 84.9% had at least one 30-day mental disorder (sTable 3).

Age was significantly associated with both 12-month and 30-day mental disorders, with younger (18–34 years) and middle-aged (35–49 years) adults exhibiting higher odds compared to those aged 65 years and above (Table 4). Compared to married individuals, never and previously married respondents had greater odds of having a 12-month mental disorder. Respondents in the lowest income groups had elevated risks of having a mental disorder in the past 30 days compared to those in the high-income group. Additionally, individuals with comorbid lifetime disorders showed higher persistence of 12-month disorders (Table 4). Furthermore, among respondents with any 12-month mental disorder, males, those with low education levels (primary or below), and those with three or more disorders were at higher risk of being classified as severe (Table 5).

**Table 4:** Sociodemographic correlates for 12-month and 30-day prevalence and persistence of mental disorders in the WMHHK.

	12-month mental disorder		30-day mental disorder	
	Prevalence	Persistence^a^	Prevalence	Persistence^b^
	OR (95% CI)	OR (95% CI)	OR (95% CI)	OR (95% CI)
**Sex**				
Female	1.1 (0.8–1.5)	0.9 (0.5–1.4)	1.1 (0.9–1.4)	0.7 (0.3–1.7)
Male	(Ref)	(Ref)	(Ref)	(Ref)
χ^2^_1_ (p-value)	0.8 (0.39)	0.5 (0.50)	1.6 (0.23)	0.6 (0.46)
**Age (years)**				
18–34	**1.9 (1.2**–**3.0)**	1.0 (0.4–2.4)	**2.4 (1.5**–**3.8)**	2.4 (0.6–10.2)
35–49	**1.8 (1.2**–**2.9)**	0.7 (0.4–1.3)	**2.4 (1.4**–**3.9)**	**2.6 (1.1**–**6.3)**
50–64	1.4 (0.8–2.3)	0.6 (0.2–1.3)	1.6 (0.9–2.8)	1.3 (0.4–4.1)
65 and above	(Ref)	(Ref)	(Ref)	(Ref)
χ^2^_3_ (p-value)	**5.7 (0.007)**	0.8 (0.49)	**6.9 (0.003)**	2.1 (0.14)
**Education**^c^				
Low	0.6 (0.3–1.1)	1.5 (0.7–3.0)	0.6 (0.3–1.3)	0.4 (0.1–3.1)
Low average	0.9 (0.6–1.5)	1.6 (0.8–3.2)	1.1 (0.5–2.3)	1.0 (0.2–5.2)
High average	1.2 (0.8–1.9)	1.7 (0.8–3.8)	1.1 (0.6–1.9)	0.4 (0.1–1.8)
High	(Ref)	(Ref)	(Ref)	(Ref)
χ^2^_3_ (p-value)	3.1 (0.05)	0.7 (0.59)	1.6 (0.22)	2.2 (0.13)
**Income**^d^				
Low	1.3 (0.8–2.0)	1.7 (0.8–3.6)	**1.7 (1.1**–**2.5)**	2.9 (0.6–14.6)
Low average	1.2 (0.8–1.9)	1.5 (0.9–2.6)	1.4 (0.8–2.4)	2.0 (0.6–6.2)
High average	1.1 (0.7–1.6)	1.1 (0.5–2.4)	1.2 (0.7–2.0)	1.5 (0.5–4.1)
High	(Ref)	(Ref)	(Ref)	(Ref)
χ^2^_3_ (p-value)	0.7 (0.59)	1.8 (0.18)	2.4 (0.11)	0.6 (0.63)
**Employment status**				
Others^e^	1.5 (1.0–2.2)	1.4 (0.7–2.7)	1.4 (0.9–2.2)	0.8 (0.3–2.3)
Working	(Ref)	(Ref)	(Ref)	(Ref)
χ^2^_1_ (p-value)	4.4 (0.05)	0.9 (0.35)	2.0 (0.17)	0.2 (0.67)
**Marital status**				
Never married	**1.8 (1.2**–**2.6)**	1.2 (0.6–2.7)	1.6 (1.0–2.6)	0.5 (0.2–1.9)
Previously married^f^	**1.8 (1.1**–**2.8)**	1.1 (0.5–2.1)	**1.7 (1.0**–**2.7)**	0.9 (0.4–2.3)
Married	(Ref)	(Ref)	(Ref)	(Ref)
χ^2^_2_ (p-value)	**9.3 (0.002)**	0.1 (0.88)	**3.9 (0.040)**	0.8 (0.48)
**Number of 12-month disorders**				
Three or more	–	–	–	**45.5 (9.4**–**219.2)**
Two	–	–	–	**9.4 (3.8**–**23.6)**
One	–	–	–	(Ref)
χ^2^_2_ (p-value)	–	–	–	**40.3 (<0.001)**
**Number of lifetime disorders**				
Three or more	–	**8.7 (4.8**–**15.8)**	–	–
Two	–	**2.5 (1.6**–**3.9)**	–	–
One	–	(Ref)	–	–
χ^2^_2_ (p-value)	–	**38.0 (<0.001)**	–	–
**Total model**				
χ^2^_13/15_ (p-value)	3.9 (0.05)	**40.5 (0.001)**	**10.6 (0.004)**	**6.7 (0.040)**

OR, odds ratio; CI, confidence interval. Bolded values indicate statistical significance at the 0.05 level, two-sided test.

a The 12-month persistence is calculated as the 12-month prevalence among lifetime cases; the significant correlates remain after removing “number of lifetime disorders” from the model.

b The 30-day persistence is calculated as the 30-day prevalence among 12-month cases; the significant correlates remain after removing “number of 12-month disorders” from the model.

c Educational attainment is defined as low (primary education or below), low-average (secondary education or post-secondary diploma), high-average (associate degree or bachelor's degree), and high (master's degree or above).

d Income is categorised into low, low-average, high-average, and high, based on the ratio of income per capita to the median income (low defined as less than half the median, low-average as up to the median, high-average as up to two times the median, and high as more than two times the median).

e Others include students, homemakers, retirees and others.

f Previously married includes separated, divorced and widowed individuals.

**Table 5:** Sociodemographic correlates for 12-month and 30-day severe mental disorders in the WMHHK.

	12-month severe mental disorder		30-day severe mental disorder	
	Prevalence	Among 12-month cases^a^	Prevalence	Among 30-day cases^b^
	OR (95% CI)	OR (95% CI)	OR (95% CI)	OR (95% CI)
**Sex**				
Female	0.8 (0.5–1.1)	**0.5 (0.3**–**0.7)**	0.8 (0.5–1.2)	0.5 (0.2–1.0)
Male	(Ref)	(Ref)	(Ref)	(Ref)
χ^2^_1_ (p-value)	2.4 (0.14)	**11.1 (0.004)**	1.6 (0.22)	3.9 (0.06)
**Age (years)**				
18–34	2.7 (0.6–11.7)	1.6 (0.2–10.6)	2.9 (0.6–15.0)	0.9 (0.1–6.4)
35–49	2.6 (0.8–7.9)	1.5 (0.3–7.7)	3.6 (0.8–15.5)	1.2 (0.2–7.1)
50–64	1.9 (0.5–6.9)	1.2 (0.2–7.5)	2.1 (0.4–10.4)	0.8 (0.1–5.0)
65 and above	(Ref)	(Ref)	(Ref)	(Ref)
χ^2^_3_ (p-value)	1.3 (0.32)	0.2 (0.93)	1.7 (0.20)	0.3 (0.83)
**Education**^c^				
Low	2.7 (0.8–9.9)	**5.2 (1.0**–**31.2)**	2.4 (0.5–12.2)	4.0 (0.5–34.1)
Low average	**2.8 (1.1**–**6.8)**	2.6 (0.6–12.1)	2.5 (0.8–8.2)	1.9 (0.2–15.3)
High average	2.0 (0.8–5.0)	1.3 (0.3–6.1)	1.9 (0.6–5.9)	1.3 (0.2–11.5)
High	(Ref)	(Ref)	(Ref)	(Ref)
χ^2^_3_ (p-value)	1.8 (0.19)	1.7 (0.22)	0.9 (0.49)	0.6 (0.64)
**Income**^d^				
Low	1.4 (0.6–3.5)	0.8 (0.2–2.9)	1.9 (0.8–4.4)	0.9 (0.2–3.4)
Low average	1.5 (0.6–3.8)	1.5 (0.3–6.3)	1.4 (0.5–3.7)	1.4 (0.3–5.5)
High average	1.1 (0.4–3.0)	1.1 (0.4–2.7)	1.2 (0.5–3.1)	1.0 (0.4–2.5)
High	(Ref)	(Ref)	(Ref)	(Ref)
χ^2^_3_ (p-value)	0.5 (0.67)	0.7 (0.59)	1.6 (0.23)	0.2 (0.89)
**Employment status**				
Others^e^	1.7 (1.0–2.9)	1.5 (0.6–4.0)	1.7 (1.0–3.1)	1.6 (0.5–5.5)
Working	(Ref)	(Ref)	(Ref)	(Ref)
χ^2^_1_ (p-value)	3.8 (0.07)	0.7 (0.41)	4.3 (0.05)	0.6 (0.44)
**Marital status**				
Never married	1.8 (0.8–3.9)	1.0 (0.3–3.1)	1.8 (0.8–4.3)	1.5 (0.4–5.1)
Previously married^f^	2.2 (0.9–5.8)	1.5 (0.5–4.8)	2.4 (0.9–6.6)	2.0 (0.6–6.3)
Married	(Ref)	(Ref)	(Ref)	(Ref)
χ^2^_2_ (p-value)	1.8 (0.20)	0.3 (0.75)	1.8 (0.19)	0.7 (0.49)
**Number of 30-day disorders**				
Three or more	–	–	–	**11.1 (4.4**–**27.8)**
Two	–	–	–	1.9 (0.8–4.3)
One	–	–	–	(Ref)
χ^2^_2_ (p-value)	–	–	–	**15.0 (<0.001)**
**Number of 12-month disorders**				
Three or more	–	**5.1 (2.3**–**11.1)**	–	–
Two	–	3.3 (0.9–11.5)	–	–
One	–	(Ref)	–	–
χ^2^_2_ (p-value)	–	**9.3 (0.002)**	–	–
**Total model**				
χ^2^_13/15_ (p-value)	**28.3 (<0.001)**	**18.1 (0.006)**	**19.8 (<0.001)**	5.2 (0.06)

OR, odds ratio; CI, confidence interval. Bolded values indicate statistical significance at the 0.05 level, two-sided test.

a The significant correlates remain after removing “number of 12-month disorders” from the model.

b The significant correlates remain after removing “number of 30-day disorders” from the model.

c Educational attainment is defined as low (primary education or below), low-average (secondary education or post-secondary diploma), high-average (associate degree or bachelor's degree), and high (master's degree or above).

d Income is categorised into low, low-average, high-average, and high, based on the ratio of income per capita to the median income (low defined as less than half the median, low-average as up to the median, high-average as up to two times the median, and high as more than two times the median).

e Others include students, homemakers, retirees and others.

f Previously married includes separated, divorced and widowed individuals.

Anxiety disorders were significantly more prevalent among females, middle-aged individuals (35–49 years), and those who were not married. Mood disorders were more prevalent among individuals under 65 years old, in the lowest income group, those who were not working, and those who were never married. Externalising disorders were more prevalent among males (sTable 4). PTSD was more prevalent among middle-aged adults, those in the lowest income group, and those previously married, whereas OCRD was more prevalent among women and those never married (sTable 5).

## Discussion

This population-based cohort study is among the first WMH-CIDI surveys in the past decade to provide representative estimates of 12-month and 30-day prevalence of mental disorders in a dynamic, rapidly changing Asian region affected by major population events. We assessed a broad range of mental disorders, capturing not only their prevalence but also their persistence and severity in the general population, utilising the latest DSM-5 criteria. Our findings showed a substantial mental health burden in the COVID-19 endemic and post-pandemic period, with one in ten (10.6%) adults having at least one mental disorder in the past 12 months, and 28.5% of these cases classified as severe. Anxiety disorders were the most prevalent condition (8.0%), followed by mood disorders (4.3%) and externalising disorders (1.7%). Notably, half of the respondents with lifetime mental disorders showed persistence of symptoms in the past 12 months, suggesting substantial chronicity of mental disorders in this population.

Our 30-day prevalence estimates for other anxiety disorders (including panic disorder, PTSD, and OCRD) were higher than the 1-week prevalence reported by the HKMMS over a decade ago (5.3% vs. 1.5%).^18^ PTSD was one of the most prevalent disorders in this study, aligning with our previous findings that reported a prevalence of suspected PTSD at 12.8% during the 2019 social unrest.^11^ This likely reflects societal shifts and recent stressors in Hong Kong, which might have increased psychological distress within the community, highlighting the significant mental health impact of these societal upheavals.^31^ In contrast, the prevalence of MDD and GAD in our data was lower compared to HKMMS (MDD: 1.1% vs. 2.9%, GAD: 1.7% vs. 4.2%). The prevalence of depression and anxiety in our study did not appear as high as in other local COVID-19 studies,^32^, ^33^, ^34^ which were primarily conducted during early pandemic phases using telephone or web-based surveys with non-probabilistic sampling and non-diagnostic instruments that may inflate estimates.^35^ Our study, therefore, offers a benchmark of the current mental health burden in Hong Kong. However, comparisons with HKMMS require caution due to differences in methodology (CIDI-5 vs. Revised Clinical Interview Schedule, CIS-R^36^), diagnostic criteria (DSM-5 vs. International Classification of Diseases 10th revision, ICD-10^37^), and timeframes (30-day vs. 1-week). Our study also covered a broader range of mental disorders, including PTSD and OCRD, which may explain the higher prevalence while providing a more comprehensive understanding of mental health amid recent societal stressors.

Our findings revealed that the 12-month prevalence of mental disorders in Hong Kong were generally lower than that reported in studies from the other two surveys that have so far reported results based on CIDI-5, Norway and Qatar.^8^^,^^9^ These differences may be attributed to the timing of surveys: Norway's was conducted during the first six months of the COVID-19 pandemic (January to September 2020), and Qatar's during the first and second waves (2021–2022), periods likely associated with heightened mental health issues. In contrast, the WMHHK took place during the endemic and post-pandemic period (2022–2024), when pandemic stress may have lessened. Conversely, our study reported higher 12-month prevalence of anxiety and depressive disorders compared to those reported based on surveys using CIDI 3.0 in other Asian regions, including mainland China, Singapore, Korea, and Japan, that were surveyed before the pandemic (2011–2016).^38^, ^39^, ^40^, ^41^ Specifically, we observed higher rates of GAD (2.1% vs. 0.2–1.0%), OCD (3.7% vs. 1.6–2.9%), and persistent depressive disorder (2.0% vs. 0.2–1.0%). Similarly, our MDD prevalence (2.8%) was comparable to that of Korea (3.1%) and Japan (2.7%), but slightly higher than mainland China (2.1%) and Singapore (2.3%). Although these regions share similar urban stressors (e.g., high density, socioeconomic pressures), differences in the social contexts may explain the higher prevalence of anxiety and depressive disorders in Hong Kong. Recent population events, such as prolonged social unrest, might have contributed to elevated and persistent levels of anxiety and stress.^42^^,^^43^ These effects are compounded by environmental, systemic, and cultural factors, as well as variations in mental health awareness, stigma, and access to care.^44^^,^^45^ These findings underscore the need for tailored mental health interventions that address risk factors associated with Hong Kong's social context, while drawing on relevant approaches from comparable settings such as Singapore.^46^, ^47^, ^48^

We reported a high persistence (12-month/lifetime prevalence ratio) of mental disorders (49.0%). BPS, OCRD, panic disorder, IED, and GAD were among conditions showing notably high persistence (52.3–64.2%). Our findings underscore the profound impact of consecutive population events that may have substantially affected population well-being. Social unrest with major protests and episodes of violence over recent years has created a persistent environment of stress, uncertainty, and trauma within the city.^11^ This has been compounded by the COVID-19 pandemic, causing prolonged social isolation, disrupted daily routines, and economic instability.^49^ These chronic stressors can sustain emotional distress and symptom severity, further impede recovery and prolong mental disorders. The cumulative effect of population events has thus played a significant role in the notably high persistence rates observed in Hong Kong.^50^ Additionally, compared to earlier local studies, higher persistence reflects the inherent clinical profiles of these disorders, which often involve recurrent mood episodes or patterns resistant to short-term resolution, persisting over a lifetime without full remission.^51^^,^^52^ Systemic barriers, such as cultural stigma and limited access to mental health services in Hong Kong,^53^ may further contribute to the high persistence rates. This highlights the urgent need for tailored interventions addressing both clinical characteristics and contextual factors−such as population stressors−that drive chronicity of the mental disorders.^54^

Furthermore, our findings on sociodemographic correlates identified potentially vulnerable groups in Hong Kong during the post-pandemic period. Younger and middle-aged adults were associated with 12-month and 30-day mental disorders, with the middle-aged group at higher risk for anxiety and mood disorders. These trends can be attributed to stressors such as economic instability and future uncertainties that impact youth,^55^ as well as financial pressures, caregiving responsibilities, and workplace burdens affecting middle-aged individuals in Hong Kong's high-stress urban environment.^14^ Also, COVID-19 physical distancing measures have significantly impacted social isolation and loneliness.^12^ Alternatively, low education levels were linked to severe mental disorders, broadly consistent with existing research that limited education may increase susceptibility to severe mental health issues due to lower health literacy, restricted access to resources, and greater exposure to socioeconomic stressors.^56^ Despite Hong Kong's compulsory education system,^57^ some individuals may still attain limited education due to financial or personal constraints. Such economic instability can increase financial stress and impede access to timely mental health care, thereby worsening their conditions. Lastly, never or previously married individuals had a higher risk of 12-month mental disorders, particularly anxiety, which points to the potential impact of social isolation and societal pressure in Chinese cultural norms of marriage.^58^ Collectively, more attention should be paid to these socially disadvantaged groups.

To address the observed burden and persistence of mental disorders, coordinated, evidence-informed actions are needed across all levels. At the population level, promoting media literacy and digital well-being by reducing excessive social media exposure during crises can alleviate stress and rumination.^11^^,^^13^^,^^59^ Citywide initiatives that foster social connection, encourage physical activity, and leverage digital mental health tools can build resilience.^60^^,^^61^ Community-based interventions, such as workplace mental health programmes and initiatives that enhance social connection and mental health literacy, can mitigate stress from economic uncertainty and loneliness among vulnerable groups.^62^, ^63^, ^64^ Labour and social protection policies, including job search assistance, skills training, and subsidised employment, can buffer the mental health impacts of unemployment and economic instability.^65^ In clinical care, tailored psychological treatments, such as exposure and response prevention, should target disorder-specific symptoms, while secondary and tertiary prevention, including anti-stigma campaigns, routine screening with clear referral pathways, and relapse-prevention planning, are critical for timely intervention and minimising symptom chronicity.^60^^,^^66^ Integrating mental health services into primary care through task-sharing with trained non-specialists, expanding guided self-help interventions, and ensuring equitable insurance coverage can reduce financial barriers, close access gaps, and deliver a comprehensive approach to care.^60^^,^^67^

Several limitations of this study warrant consideration. First, methodological differences in diagnostic instruments, sampling strategies, and recall periods (e.g., 12-month vs. lifetime) limit direct comparisons with other epidemiologic studies in Chinese populations. Second, although we employed standardised diagnostic criteria, the reliance on self-reported symptoms potentially introduces recall bias and underreporting, particularly for stigmatised conditions like substance use disorders. Nevertheless, the high concordance with clinical interviews reported in our study demonstrates the good clinical validity of the CIDI-5. Of note, the zero prevalence of SUD in our study may also reflect the impact of stringent COVID-19 measures, including traveller quarantines, restrictions to high-risk areas, and trade inspections, which curbed the import and circulation of illegal drugs.^68^ Third, the cross-sectional design precludes causal interpretation of the observed associations. Despite this, our study identified vulnerable groups that can guide prevention and intervention strategies. Fourth, we did not assess psychotic or personality disorders. These disorders are less prevalent in community samples and generally require clinical assessments for valid case identification. Future population-based studies should incorporate feasible and validated assessments, such as two-stage designs using screening tools followed by clinician diagnoses, for these disorders.^22^, ^23^, ^24^, ^25^ Fifth, while our sample was population-representative of the household population, it excluded specific subgroups (e.g., institutionalised or homeless populations) and non-Chinese speaking individuals. Finally, although overall response and cooperation rates were modest, they were comparable to those of other surveys conducted during the pandemic.^8^^,^^9^ Nevertheless, we implemented appropriate weighting procedures to address potential bias and enhance representativeness, alongside a multi-contact protocol and flexible scheduling and interview modes to reduce access barriers and optimise recruitment. Subsequent phases will expand recruitment to increase sample size, improving precision for less prevalent disorders.

In conclusion, this study provides unprecedented population-representative data, revealing that 1 in 10 adults in Hong Kong had a mental disorder in the past 12 months. Utilising the gold-standard CIDI-5, we ensure diagnostic accuracy for internationally comparable DSM-5 disorders, with data from the late COVID-19 period offering the latest snapshot of post-pandemic mental health. Our findings underscore a significant mental health burden, characterised by substantial persistence and severity, urging tailored interventions to address Hong Kong's urban stressors, systemic barriers, and the impact of major population events. It also emphasises the need to enhance mental health services for vulnerable groups like younger and middle-aged adults, less educated, or unmarried individuals. Implementing a three-tier public mental health framework, comprising primary prevention, secondary intervention, and tertiary care, can effectively address these needs.^69^ Multifaceted interventions involving collaborative efforts from government, policymakers, health and social care sectors, and philanthropies are vital. Future monitoring of prevalence and longitudinal research is important to elucidate underlying risk factors and inform sustainable public health strategies to enhance population mental health.

## Contributors

MYN conceived the study. CSMW and MYN designed the study. CSMW, CMCL, XX, SBKW, WCC1, WCC2, NXY, CPWC, AKKC, EHML, and MYN carried out the study. CSMW, SW, FPF, XX, and WTC analysed the data. CSMW, CMCL, YK, WGA, RCK, and MYN interpreted the data. CSMW, CMCL, and MYN wrote the first draft of the manuscript. MYN acquired funding. All authors critically revised the manuscript and approved the final version.

## Data sharing statement

Deidentified participant data and data dictionary can be obtained upon request made to Dr Michael Y Ni (nimy@hku.hk), the corresponding author of the study.

## Declaration of interests

In the past 3 years, Ronald C Kessler was a consultant for Cambridge Health Alliance, Child Mind Institute, Massachusetts General Hospital, RallyPoint Networks, Inc., Sage Therapeutics, University of Michigan, and University of North Carolina. He has stock options in Cerebral Inc., Mirah, PYM (Prepare Your Mind), and Verisense Health. He owns an interest in Menssano LLC.
