# Supplementary material for: Prevalence, persistence, and severity of 12-month and 30-day DSM-5 disorders in the World Mental Health Hong Kong Study
Source: Lancet Reg Health West Pac. 2025 Nov 26;65:101757. doi: 10.1016/j.lanwpc.2025.101757 (PMC12689180; doi:10.1016/j.lanwpc.2025.101757)
Supplement: Supplementary Tables [file mmc1.docx]

**Supplementary appendix**

**Table of Contents**

[Supplementary methods 2](#_Toc214570469)

[sTable 1. Concordance testing of CIDI-5 against the Structured Clinical Interview for DSM-5 (SCID-5). 5](#_Toc214570470)

[sTable 2. Summary of the CIDI-5 modules in WMHHK. 6](#_Toc214570471)

[sTable 3. Twelve-month and 30-day DSM-5 disorders distribution by severity in the WMHHK. 7](#_Toc214570472)

[sTable 4. Sociodemographic correlates for 12-month DSM-5 anxiety, mood, and externalising disorders in the WMHHK. 8](#_Toc214570473)

[sTable 5. Sociodemographic correlates for 12-month DSM-5 PTSD, OCRD, and other anxiety disorders in the WMHHK. 9](#_Toc214570474)

# Supplementary methods

**Background and objectives**

Granted permission to develop and translate the Traditional Chinese Composite International Diagnostic Interview-5 (CIDI-5), the FAMILY Cohort team at The University of Hong Kong began the instrument development process in early 2019. The initial forward translation was completed in late 2020, followed by a comprehensive review conducted by an expert panel. To ensure the translation’s validity and equivalence with the original CIDI-5, the team implemented cognitive interviewing techniques, which facilitated the revision of questions based on respondent feedback to enhance clarity and cultural compatibility. The revised translation was reviewed and endorsed by the expert panel in early 2021. The final stages of the process involved collaboration with the World Mental Health (WMH) Survey Initiative, Harvard Medical School, and the University of Michigan, finalising the translation and the configuration of the Blaise software for a pilot test in late 2021.

**Expert panel review, cognitive interviewing and cultural adaptation**

The Traditional Chinese Hong Kong version of the CIDI-5 was developed based on the English International Interviewer Administered (IVWR) Full 3.4 (2020 Beta Test) version. The translation process involved the adaptation of twenty CIDI-5 modules by bilingual research team members with expertise in mental health. These translations were subsequently reviewed by an expert panel comprising four psychiatrists and one psychologist to ensure alignment with the requirements of psychiatric epidemiologic surveys in Hong Kong. Cognitive interviewing was used to identify ambiguous and cognitively challenging aspects of question wording, structure, and delivery by qualitatively analysing verbal responses. Between November and December 2020, a total of 30 cognitive interviews (one in-person and others via online or telephone interviews due to COVID-19 restrictions) were conducted. All sessions were audio-recorded. Two interviewers each completed 15 interviews, while one observer coded all sessions. The interviewers utilised mixed methods, incorporating “think-aloud” and neutral probing techniques, particularly for questions where respondents exhibited confusion. This approach uncovered significant comprehension challenges and highlighted the need for cultural adaptation (e.g., using simplified language for complex terms and providing contextualised examples that reflect the Hong Kong context). A coding scheme was developed to systematically classify response issues (e.g., comprehension difficulties, ambiguity, and recall problems). The observer documented coded responses, creating a dataset for qualitative analysis. Regular interviewer-observer meetings were held to review findings, propose alternative translations, and calibrate judgments to enhance reliability. Decisions regarding revisions, retention of original translations, or further consultation with the expert panel or the WMH team were made through unanimous agreement among interviewers and observers.

**Pilot testing**

Fieldworker recruitment (4 interviewers and 1 supervisor) was facilitated by an external survey company in August 2021. The interviewer training (see Training section below) was led by the FAMILY Cohort team members who had completed the WMH-CIDI training program. Pilot participants were randomly selected from the FAMILY cohort, with the following inclusion criteria: (1) Hong Kong residents aged 18 or above; (2) ability to comprehend written traditional Chinese; and (3) fluency in Cantonese. Face-to-face interviews, either in-person or video-based online, were conducted with the aid of Blaise, a computer-assisted personal interviewing (CAPI) system. Each completed interview was compensated with HKD 300 (USD 38) in supermarket coupons. A total of 52 completed CIDI-5 pilot interviews were conducted between October 2021 and January 2022, with an average duration of 87 minutes. The pilot test revealed several challenges, including difficulties in participant recruitment and scheduling interviews amid the COVID-19 pandemic, a time lag between interviewer training and data collection, and potential data inaccuracies arising from input and programming errors. Despite these issues, the pilot test yielded critical insights for enhancing the CIDI-5 study, particularly in areas such as training and evaluation, field procedures, recruitment strategies, interview quality, program execution, and data management.

**Interviewer and supervisor training**

Prior to the pilot testing and main round of fieldwork, training sessions were conducted in alignment with the agenda approved by WMH. Each training cycle consisted of a 5-day general interviewer training, a 1-day supervisor training, and a 1-day evaluation assessment. The general training encompassed an overview of the project’s background and logistical framework, techniques for standardised interviewing, an overview and full run-through of each CIDI-5 module, navigation of the Blaise software, role-play exercises, and protocols for emergency response. The supervisor training focused on delineating the roles and responsibilities of supervisors, including field structure, field supervision, team management, and quality assurance procedures. Following the training sessions, all interviewers and supervisors were required to participate in a 30-minute individual evaluation. This evaluation was conducted through a role-playing exercise, wherein one trainer assumed the role of a “participant” to be interviewed, while one other trainer served as assessor. The assessment criteria included the interviewer’s comprehension of the CIDI-5 instrument, proficiency in interviewing techniques, ability to navigate Blaise for data collection, and responses to fictional emergency scenarios. All interviewers and supervisors were required to pass the evaluation before official fieldwork. Post-evaluation, individualised debriefing and feedback were provided to each interviewer to facilitate further skill enhancement. In addition, regular recap training sessions were conducted, focusing on advanced probing techniques and question reading to enhance interview fluency and ensure data quality. In cases where the initial training occurred more than one month prior to the commencement of fieldwork, interviewers and supervisors underwent retraining to refresh their interviewing skills.

**Fieldwork observation and quality control**

To evaluate the interview quality, a supervisor was assigned to observe a minimum of three entire interviews (specifically the first three) conducted by each interviewer, after obtaining participants’ consent. Supervisors were responsible for detecting and preventing falsified information, identifying issues with the interviewer-questionnaire interface, performing non-interview tasks, and providing support when necessary. The FAMILY Cohort team also deployed interviewer trainers and research assistants to observe interviews (or listen to audio recording) and provide constructive feedback to both interviewers and supervisors. Around 10% of each interviewer’s work was randomly selected for direct observation or audio recording. Follow-up meetings were regularly convened between the FAMILY Cohort team and the research directors of the survey company to monitor study progress and address challenges encountered during participant recruitment. Following the WMH Quality Assurance Guideline, regular quality checks were also implemented to ensure the reliability of the data. These checks involved monitoring various aspects of interviewers’ work, including interview duration, question reading time, daily/monthly completion rates, missing data rates, and prevalence rates per interviewer. Research assistants also followed up on unusual cases for verification.

Quality control measures were implemented by the survey company for both incomplete and completed interviews, with detailed reports subsequently submitted to the FAMILY Cohort team. For household members who could not be contacted or were deemed ineligible (e.g., deceased, institutionalised, or language barriers), approximately 5% of the sample was re-contacted by supervisors to confirm their unavailability. Around 10% of the participants who refused to join the study were re-contacted and invited to participate in an exit interview. Questions for verification included: (1) if the members had received any phone or invitation message of the study, and (2) the reason for their refusal to participate. For completed interviews, approximately 10% of the sample was re-contacted by supervisors for verification purposes. Questions for verification included: (1) the date and time of the interview, (2) the approximate duration of the interview, (3) a subjective rating of the interviewer’s professionalism, and (4) the coupons of which supermarket and the amount received. Additionally, demographic verification questions included: (5) the highest level of education attained, (6) place of birth, (7) the presence of any chronic physical health conditions, (8) the specific name(s) of the physical health condition(s), (9) the total number of household members, and (10) the number of household members under the age of 18. These measures were implemented to ensure data accuracy and uphold the integrity of the study.

**Fieldwork recruitment**

The fieldwork recruitment commenced in November 2022, with advanced invitation letters distributed to all eligible households. These letters provided comprehensive information regarding study background, objectives, procedures, potential costs and benefits, incentives, voluntary participation and withdrawal policies, the use of personal data, confidentiality assurances, and contact details of the research team. To optimise recruitment, invitations were disseminated in multiple rounds, followed by reminders via phone calls, SMS messages, and email invitations (where applicable). As a token of appreciation for their participation, respondents were offered supermarket coupons valued at HKD 500 (USD 64). A team of 42 interviewers and 4 supervisors was recruited to conduct the main round study. The interviewers worked flexible daily shifts between 9 a.m. and 9 p.m., including both weekdays and weekends, to accommodate respondents’ availability. All interviews were conducted face-to-face in real-time by trained interviewers in two modes: in-person or online video-based. Although the instrument was initially designed for in-person interviews, the methodology was adapted to include online video-based interviews in response to constraints imposed by the COVID-19 pandemic. Most of the interviews were conducted via online video, with the option to conduct in-person interviews if respondents were unable or unwilling to participate online. Scheduling was coordinated via phone calls, SMS, or email, with reminders sent 24-48 hours prior. For online interviews, respondents received a unique meeting link and brief technical guidance for video-based interviews. For in-person interviews, supervisors confirmed the address, access, and COVID-19 infection control requirements. Both modes used the same Blaise CAPI instrument, content, and skip logic. To facilitate the interview process, respondent booklets were either mailed or shared electronically, enabling participants to review the material during the interview. To facilitate the interview process, respondent booklets were either mailed or shared electronically, enabling participants to review the material during the interview. Data collection spanned 17 months, from November 2022 to March 2024, during which 3,053 participants were successfully recruited. The average interview duration was 89 minutes. To ensure data quality and consistency, interviewers and supervisors underwent rigorous training, and comprehensive quality control measures were implemented, as detailed in the methodology earlier.

**Weighting and survey design**

The final sampling weights were constructed in two stages to address potential attrition bias and enhance sample representativeness. First, we implemented inverse probability weighting (IPW), using a logistic regression to predict the probability of participation in the WMHHK based on baseline FAMILY Cohort covariates, including sex, age, educational level, marital status, household income, housing type, and employment status. The IPW weights were calculated as the inverse of the predicted participation probabilities. Second, we calibrated the IPW weights to the general population via post-stratification raking to the Hong Kong Population Census margins for sex, age, educational level, marital status, household income, and housing type, yielding the final sampling weights.

**Sensitivity analysis**

To evaluate the potential effects of interview mode, we conducted sensitivity analyses using machine-learning models for robust confounding adjustment. We trained separate random forest models for each 12‑month diagnostic outcome (any mental disorder, three disorder categories, and seven individual disorders; bipolar spectrum disorder, intermittent explosive disorder, and substance use disorder were excluded due to prevalence below 1%) using all available sociodemographic covariates (sex, age, marital status, employment status, occupation, education level, parental education level, income, born in Hong Kong) to generate respondent‑level predicted probabilities (risk scores). We then fitted logistic regression models that included the risk score and interview mode (in-person vs. online video) to test whether interview mode was associated with each outcome, conditional on covariate-informed risk. Interview mode was not significantly associated with any 12‑month disorder outcomes (p > 0.05).

**Definition of severity**

Respondents were categorised as having a *severe* mental disorder if they met criteria for: (1) 12‐month bipolar I disorder, or (2) 12-month alcohol use disorder/substance use disorder with physiological symptoms, or (3) suicide attempt in the past 12 months and at least one 12-month core disorder (bipolar spectrum disorders, alcohol use disorder, substance use disorder, major depressive episode (MDE), generalised anxiety disorder (GAD), post-traumatic stress disorder (PTSD), persistent depressive disorder, obsessive-compulsive disorder, panic disorder, or intermittent explosive disorder), or (4) at least one 12-month core disorder with very high 12‐month disorder‐related interference, or (5) 12-month MDE, GAD, or PTSD with very high 30-day MDE/GAD/PTSD interference or distress, or (6) at least one 12-month core disorder with high or very high 30-day interference in two or more areas of life (home, work, relationships, or social life).

Respondents were categorised as *moderate* if they were not severe and met criteria for: (1) at least one 12-month core disorder with moderate or high 12-month disorder‐related interference, or (2) 12-month MDE, GAD, or PTSD with moderate or high 30-day MDE/GAD/PTSD interference or distress, or (3) at least one 12-month core disorder with moderate or high or very high 30-day interference in one or more areas of life (home, work, relationships, or social life).

Respondents were categorised as *mild* if they were not severe/moderate and met criteria for: (1) at least one 12-month core disorder with no or mild 12-month disorder‐related interference, or (2) 12-month MDE, GAD, or PTSD with no or mild 30-day MDE/GAD/PTSD interference or distress, or (3) at least one 12-month core disorder with no or mild 30-day interference in one or more areas of life (home, work, relationships, or social life), or (4) none of the above condition were met and at least one 12-month disorder (core and non-core disorders, including excoriation, trichotillomania, hoarding disorder, body dysmorphic disorder, and ADHD).

# sTable 1. Concordance testing of CIDI-5 against the Structured Clinical Interview for DSM-5 (SCID-5).

| **Diagnosis** | **Sensitivity** | **Specificity** | **PPV** | **NPV** | **TCA** | **AUC** |
| --- | --- | --- | --- | --- | --- | --- |
| Major depressive disorder | 78.8 | 97.3 | 92.2 | 91.3 | 91.7 | 88.1 |
| Generalised anxiety disorder | 78.8 | 93.3 | 83.9 | 90.9 | 88.9 | 86.1 |
| Post-traumatic stress disorder | 85.7 | 78.2 | 51.4 | 98.8 | 78.7 | 82.0 |

PPV, positive predictive value; NPV, negative predictive value; TCA, total classification accuracy; AUC, area under the receiver operator characteristics (ROC) curve.

# sTable 2. Summary of the CIDI-5 modules in WMHHK.

| **Section** | **Module** | **Diagnosis** |
| --- | --- | --- |
| Background | BA: Your background | -- |
|  | HE: Your health | -- |
| Diagnostic module | DE: Depression | Major depressive disorder (MDD) |
|  | PD: Persistent depression | Persistent depressive disorder (PDD) |
|  | SH: Self-harm | Suicidal ideation, plan, attempt, and gesture;  Non-suicidal self-injury (NSSI) |
|  | HM: High mood | Bipolar spectrum disorder (BPS) |
|  | WA: Worry and anxiety | Generalised anxiety disorder (GAD) |
|  | AA: Anger attacks | Intermittent explosive disorder (IED) |
|  | PA: Panic attacks | Panic disorder |
|  | OC: Obsessions and compulsions | Obsessive-compulsive disorder (OCD), excoriation, trichotillomania, hoarding disorder, body dysmorphic disorder |
|  | SE: Stressful experiences | Post-traumatic stress disorder (PTSD) |
|  | TAD: Tobacco, alcohol, and drugs | Alcohol use disorder (AUD), substance use disorder (SUD) |
|  | UE: Unusual experiences | Psychotic experiences |
| Non-diagnostic module | TR: Treatment of emotional problems | -- |
|  | EM: Employment | -- |
|  | FI: Finances | -- |
|  | PR: Personal relationships | -- |
|  | SN: Social networks | -- |
|  | CE: Childhood experiences | -- |
|  | RC: Respondent contacts | -- |

# sTable 3. Twelve-month and 30-day DSM-5 disorders distribution by severity in the WMHHK.

|  | **12-month disorder^a^** | | | **30-day disorder^a^** | | |
| --- | --- | --- | --- | --- | --- | --- |
|  | **Severe %**  **(95% CI)** | **Moderate %**  **(95% CI)** | **Mild %**  **(95% CI)** | **Severe %**  **(95% CI)** | **Moderate %**  **(95% CI)** | **Mild %**  **(95% CI)** |
| Anxiety disorders |  |  |  |  |  |  |
| Panic disorder | 13.0 (7.6-18.5) | 8.9 (3.9-13.9) | 7.8 (0.1-15.5) | 8.0 (0.8-15.3) | 6.9 (2.7-11.1) | 7.2 (0.0-14.8) |
| Generalised anxiety disorder | 37.2 (23.7-50.8) | 19.1 (14.0-24.2) | 0.2 (0.0-0.7) | 35.8 (23.4-48.2) | 12.5 (7.5-17.5) | 0.0 |
| Post-traumatic stress disorder | 33.3 (20.6-45.9) | 40.7 (32.2-49.3) | 14.0 (0.6-27.4) | 27.0 (15.3-38.8) | 29.3 (23.6-34.9) | 8.6 (0.0-19.3) |
| Obsessive-compulsive and related disorders | 21.2 (11.0-31.5) | 26.3 (19.9-32.6) | 66.7 (55.9-77.4) | 18.5 (10.4-26.5) | 23.9 (18.0-29.7) | 53.3 (42.8-63.8) |
| Any anxiety disorder | 67.6 (49.2-86.1) | 74.8 (66.8-82.7) | 84.7 (72.0-97.4) | 58.4 (44.4-72.3) | 58.0 (50.2-65.8) | 66.0 (51.5-80.5) |
| Mood disorders |  |  |  |  |  |  |
| Major depressive disorder | 41.3 (27.2-55.4) | 30.5 (22.6-38.4) | 3.3 (0.0-8.1) | 25.2 (12.1-38.4) | 6.6 (1.8-11.4) | 0.0 |
| Persistent depressive disorder | 20.9 (11.7-30.0) | 24.8 (12.0-37.6) | 6.0 (0.0-14.9) | 16.7 (6.7-26.6) | 11.4 (2.6-20.3) | 0.0 |
| Bipolar spectrum disorders | 8.4 (0.1-16.7) | 4.4 (0.4-8.4) | 1.7 (0.0-5.4) | 7.7 (0.0-15.8) | 0.4 (0.0-1.3) | 1.7 (0.0-5.4) |
| Any mood disorder | 54.3 (44.5-64.2) | 47.5 (36.4-58.7) | 11.0 (0.4-21.6) | 37.9 (28.6-47.2) | 15.7 (6.0-25.3) | 1.7 (0.0-5.4) |
| Externalising disorders^b^ |  |  |  |  |  |  |
| Intermittent explosive disorder | 6.2 (2.3-10.1) | 6.4 (2.6-10.3) | 0.0 | 5.6 (1.7-9.5) | 3.3 (0.9-5.6) | 0.0 |
| Alcohol use disorder | 27.9 (15.5-40.3) | 4.0 (0.0-8.4) | 4.2 (0.0-9.1) | 22.0 (10.3-33.8) | 3.1 (0.0-7.3) | 4.2 (0.0-9.1) |
| Any externalising disorder | 34.1 (19.4-48.7) | 10.4 (4.4-16.5) | 4.2 (0.0-9.1) | 27.6 (14.1-41.1) | 6.4 (1.0-11.8) | 4.2 (0.0-9.1) |
| Number of disorders |  |  |  |  |  |  |
| One disorder | 42.4 (27.2-57.5) | 56.7 (48.4-65.0) | 96.8 (92.7-100.0) | 42.7 (28.8-56.5) | 43.6 (32.0-55.1) | 69.0 (54.7-83.2) |
| Two disorders | 28.4 (16.3-40.6) | 25.9 (17.0-34.8) | 2.5 (0.0-6.3) | 21.0 (12.6-29.4) | 20.3 (12.8-27.9) | 3.0 (0.0-7.0) |
| Three or more disorders | 29.2 (19.8-38.7) | 17.5 (10.3-24.6) | 0.7 (0.0-2.3) | 21.2 (10.7-31.8) | 4.1 (1.4-6.8) | 0.0 |
| Any mental disorder | 100.0 | 100.0 | 100.0 | 84.9 (76.7-93.0) | 68.0 (57.5-78.5) | 72.0 (57.8-86.2) |

SE, standard error.

^a^ Percentages indicate the distribution of each disorder by severity category; the unweighted frequencies of severe, moderate, and mild cases are 85, 151, and 81, respectively.

^b^ Substance use disorder is reported with zero prevalence.

# sTable 4. Sociodemographic correlates for 12-month DSM-5 anxiety, mood, and externalising disorders in the WMHHK.

|  | **12-month**  **anxiety disorder^a^** | **12-month**  **mood disorder^a^** | **12-month**  **externalising disorder^a^** |
| --- | --- | --- | --- |
|  | **OR (95% CI)** | **OR (95% CI)** | **OR (95% CI)** |
| **Sex** |  |  |  |
| Female | **1.5 (1.0-2.1)** | 1.2 (0.9-1.7) | **0.4 (0.2-0.8)** |
| Male | (Ref) | (Ref) | (Ref) |
| χ^2^_1_ (p-value) | **4.9 (0.041)** | 1.2 (0.28) | **6.6 (0.019)** |
| **Age (years)** |  |  |  |
| 18-34 | 1.6 (0.9-2.9) | **4.3 (1.1-16.3)** | 3.0 (0.5-18.8) |
| 35-49 | **1.8 (1.2-2.8)** | **4.4 (1.1-17.2)** | 1.8 (0.4-7.0) |
| 50-64 | 1.2 (0.7-2.0) | **4.2 (1.4-12.0)** | 0.7 (0.1-4.6) |
| 65 and above | (Ref) | (Ref) | (Ref) |
| χ^2^_3_ (p-value) | 3.1 (0.06) | 2.7 (0.08) | **3.8 (0.033)** |
| **Education^b^** |  |  |  |
| Low | 0.7 (0.3-1.4) | 1.2 (0.4-3.4) | 2.1 (0.4-10.9) |
| Low average | 1.2 (0.5-2.7) | 1.0 (0.5-2.2) | 1.7 (0.7-4.4) |
| High average | 1.5 (0.7-3.2) | 1.6 (0.7-3.9) | 0.9 (0.4-1.9) |
| High | (Ref) | (Ref) | (Ref) |
| χ^2^_3_ (p-value) | **4.3 (0.020)** | 1.4 (0.29) | 1.2 (0.36) |
| **Income^c^** |  |  |  |
| Low | 1.5 (1.0-2.3) | **2.1 (1.0-4.1)** | 0.3 (0.1-1.3) |
| Low average | 1.2 (0.8-2.0) | 1.0 (0.4-2.2) | 0.7 (0.3-1.5) |
| High average | 1.1 (0.6-1.8) | 1.0 (0.5-1.7) | 1.0 (0.5-2.0) |
| High | (Ref) | (Ref) | (Ref) |
| χ^2^_3_ (p-value) | 1.7 (0.22) | **4.8 (0.014)** | 1.6 (0.23) |
| **Employment status** |  |  |  |
| Others^d^ | 1.5 (0.9-2.5) | **1.7 (1.0-3.0)** | 1.0 (0.4-2.6) |
| Working | (Ref) | (Ref) | (Ref) |
| χ^2^_1_ (p-value) | 2.7 (0.12) | **4.5 (0.047)** | 0.0 (0.94) |
| **Marital status** |  |  |  |
| Never married | **1.8 (1.3-2.5)** | **2.6 (1.4-5.0)** | 1.0 (0.4-2.2) |
| Previously married^e^ | **1.8 (1.3-2.6)** | 1.8 (0.8-4.1) | 0.4 (0.1-3.4) |
| Married | (Ref) | (Ref) | (Ref) |
| χ^2^_2_ (p-value) | **10.3 (0.001)** | **5.4 (0.015)** | 0.4 (0.69) |
| **Total model**  χ^2^_13_ (p-value) | **7.0 (0.012)** | **10.7 (0.004)** | **8.3 (0.008)** |

OR, odds ratio; CI, confidence interval. Bolded values indicate statistical significance at the 0.05 level, two-sided test.

^a^ Numbers of anxiety, mood, or externalising disorders are not adjusted in models due to small sample size.

^b^ Educational attainment is defined as low (primary education or below), low-average (secondary education or post-secondary diploma), high-average (associate degree or bachelor’s degree), and high (master's degree or above).

^c^ Income is categorised into low, low-average, high-average, and high, based on the ratio of income per capita to the median income (low defined as less than half the median, low-average as up to the median, high-average as up to two times the median, and high as more than two times the median).

^d^ Others include students, homemakers, retirees and others.

^e^ Previously married includes separated, divorced and widowed individuals.

# sTable 5. Sociodemographic correlates for 12-month DSM-5 PTSD, OCRD, and other anxiety disorders in the WMHHK.

|  | **12-month**  **PTSD** | **12-month**  **OCRD** | **12-month**  **other anxiety disorders^a^** |
| --- | --- | --- | --- |
|  | **OR (95% CI)** | **OR (95% CI)** | **OR (95% CI)** |
| **Sex** |  |  |  |
| Female | 1.4 (0.8-2.3) | **1.8 (1.1-2.9)** | 1.4 (0.8-2.6) |
| Male | (Ref) | (Ref) | (Ref) |
| χ^2^_1_ (p-value) | 1.9 (0.18) | **7.4 (0.014)** | 1.3 (0.26) |
| **Age (years)** |  |  |  |
| 18-34 | 2.1 (0.9-4.7) | 0.8 (0.4-1.9) | 1.8 (0.5-7.2) |
| 35-49 | **2.5 (1.4-4.5)** | 1.3 (0.6-2.8) | 2.4 (0.7-8.3) |
| 50-64 | 1.2 (0.5-3.2) | 1.2 (0.7-2.2) | 1.5 (0.6-4.0) |
| 65 and above | (Ref) | (Ref) | (Ref) |
| χ^2^_3_ (p-value) | **5.9 (0.006)** | 0.7 (0.54) | 0.7 (0.54) |
| **Education^b^** |  |  |  |
| Low | 0.4 (0.1-1.8) | 0.7 (0.2-2.8) | 0.9 (0.2-4.7) |
| Low average | 0.9 (0.4-2.0) | 1.3 (0.4-5.1) | 1.8 (0.5-6.6) |
| High average | 1.4 (0.6-3.1) | 1.5 (0.3-6.9) | 2.9 (0.9-8.7) |
| High | (Ref) | (Ref) | (Ref) |
| χ^2^_3_ (p-value) | 1.7 (0.21) | 1.4 (0.29) | **4.3 (0.021)** |
| **Income^c^** |  |  |  |
| Low | **2.8 (1.2-6.4)** | 1.7 (0.7-4.0) | 1.2 (0.6-2.2) |
| Low average | 1.4 (0.6-3.5) | 1.4 (0.7-2.8) | 1.0 (0.5-2.2) |
| High average | 0.7 (0.4-1.4) | 1.3 (0.6-3.1) | 0.9 (0.5-1.8) |
| High | (Ref) | (Ref) | (Ref) |
| χ^2^_3_ (p-value) | **4.6 (0.016)** | 0.6 (0.61) | 0.1 (0.95) |
| **Employment status** |  |  |  |
| Others^d^ | 1.1 (0.6-2.0) | 1.3 (0.6-2.8) | 1.6 (0.6-4.1) |
| Working | (Ref) | (Ref) | (Ref) |
| χ^2^_1_ (p-value) | 0.2 (0.66) | 0.5 (0.48) | 1.2 (0.29) |
| **Marital status** |  |  |  |
| Never married | 1.4 (0.8-2.4) | **2.4 (1.6-3.5)** | 1.4 (0.5-3.5) |
| Previously married^e^ | **2.6 (1.5-4.7)** | 0.7 (0.3-1.9) | 2.2 (1.0-5.0) |
| Married | (Ref) | (Ref) | (Ref) |
| χ^2^_2_ (p-value) | **6.9 (0.006)** | **10.1 (0.001)** | 2.6 (0.11) |
| **Total model**  χ^2^_13_ (p-value) | **13.5 (0.002)** | **7.1 (0.012)** | 1.8 (0.23) |

PTSD, post-traumatic stress disorder; OCRD, obsessive-compulsive and related disorders; OR, odds ratio; CI, confidence interval. Bolded values indicate statistical significance at the 0.05 level, two-sided test.

^a^ Other anxiety disorders include panic disorder and generalised anxiety disorder.

^b^ Educational attainment is defined as low (primary education or below), low-average (secondary education or post-secondary diploma), high-average (associate degree or bachelor’s degree), and high (master's degree or above).

^c^ Income is categorised into low, low-average, high-average, and high, based on the ratio of income per capita to the median income (low defined as less than half the median, low-average as up to the median, high-average as up to two times the median, and high as more than two times the median).

^d^ Others include students, homemakers, retirees and others.

^e^ Previously married includes separated, divorced and widowed individuals.
